# Supplementary material for: Oncogenic Role of HMGB1 as An Alarming in Robust Prediction of Immunotherapy Response in Colorectal Cancer
Source: Cancers (Basel). 2022 Oct 5;14(19):4875. doi: 10.3390/cancers14194875 (PMC9563811; doi:10.3390/cancers14194875)
Supplement: Supplementary file 1 [file cancers-14-04875-s001.zip › cancers-1955337-supplementary.pdf]

cancers

MDPI

Supplementary Materials

Oncogenic Role of HMGB1 as an Alarming in Robust Prediction of Immunotherapy Response in Colorectal Cancer

Huijiao Lu, Mengyi Zhu, Lin Qu, Hongwei Shao, Rongxin Zhang and Yan Li

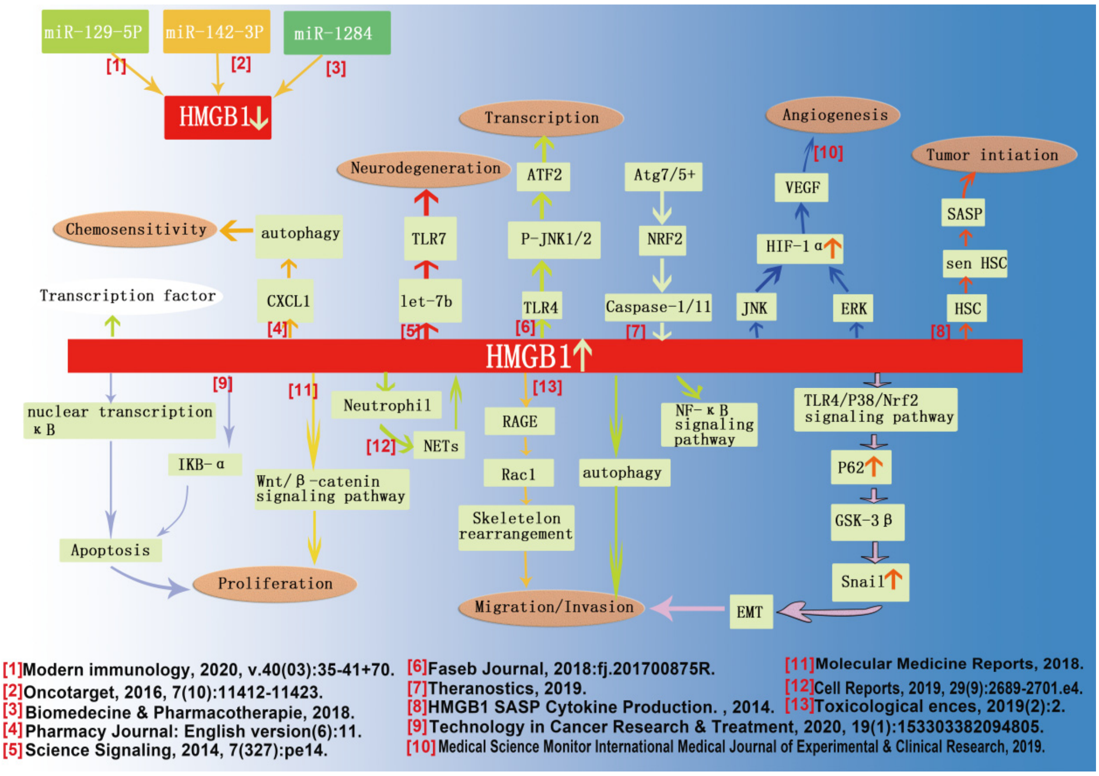

Figure S1. The diagram of HMGB1-related reported oncogenic pathway in different tumors.

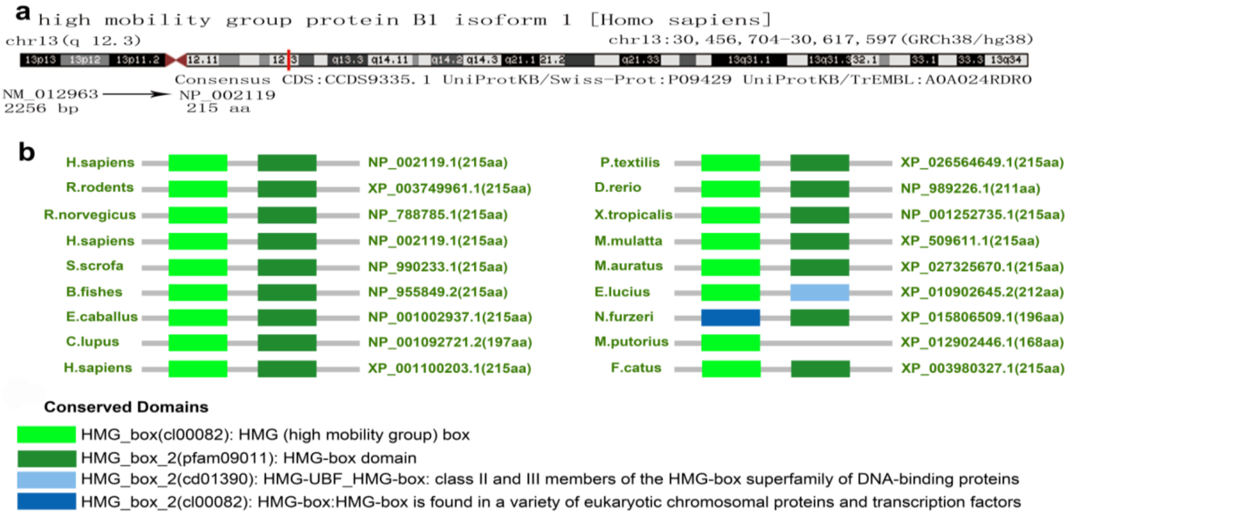

Figure S2. HMGB1-related different structural characteristics in different species. (**a**) The diagram of Genomic location of human HMGB1 (**b**) Conserved domains of HMGB1 protein in some different species.

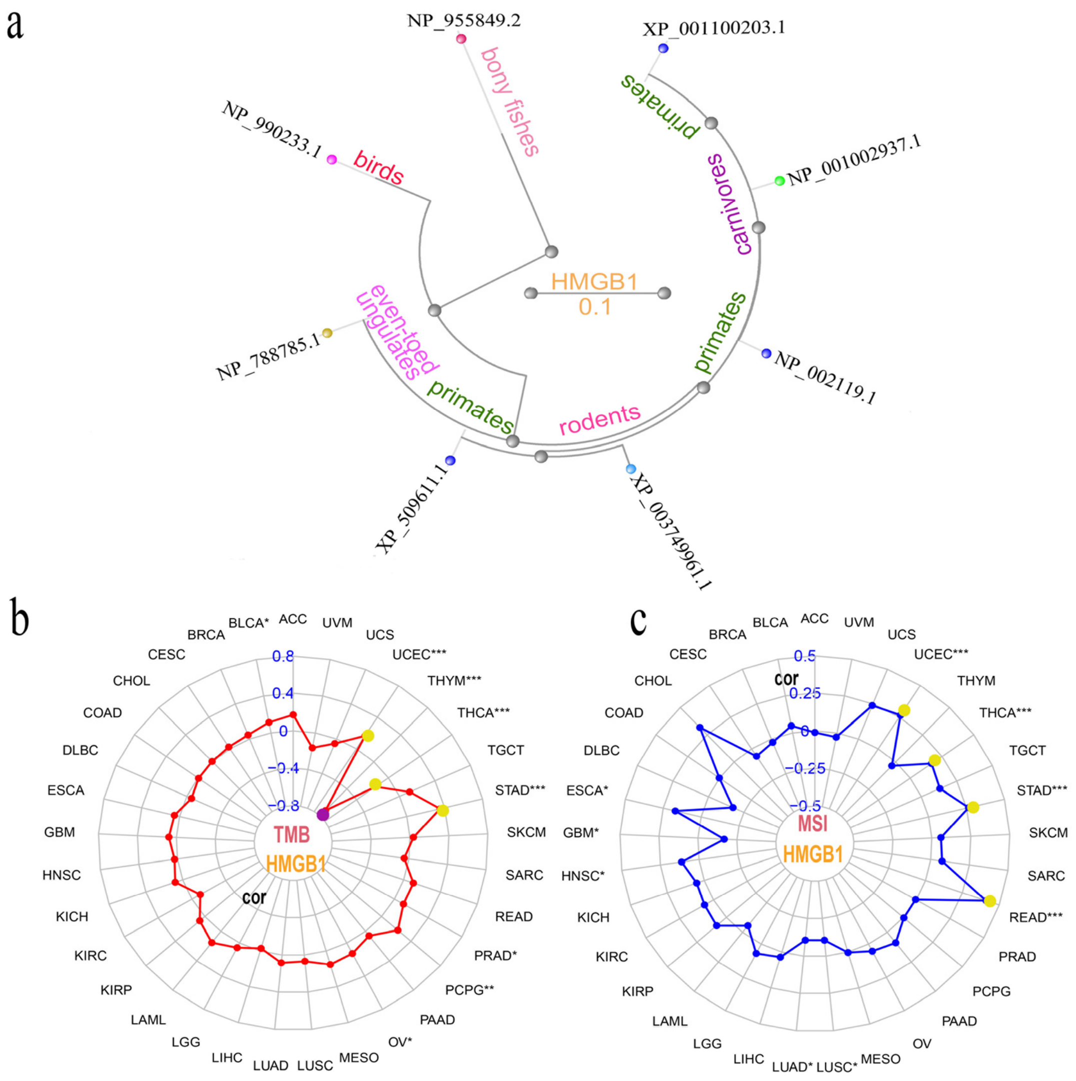

Figure S3. (a) HMGB1-related phylogenetic tree, (b) the potential correlation of HMGB1 (c) TMB/MSI. The *p*-value is given. The partial correlation (cor) values of ±0.8 (TMB) and ±0.5(MSI) are marked. * $p < 0.05$; ** $p < 0.01$; *** $p < 0.001$.

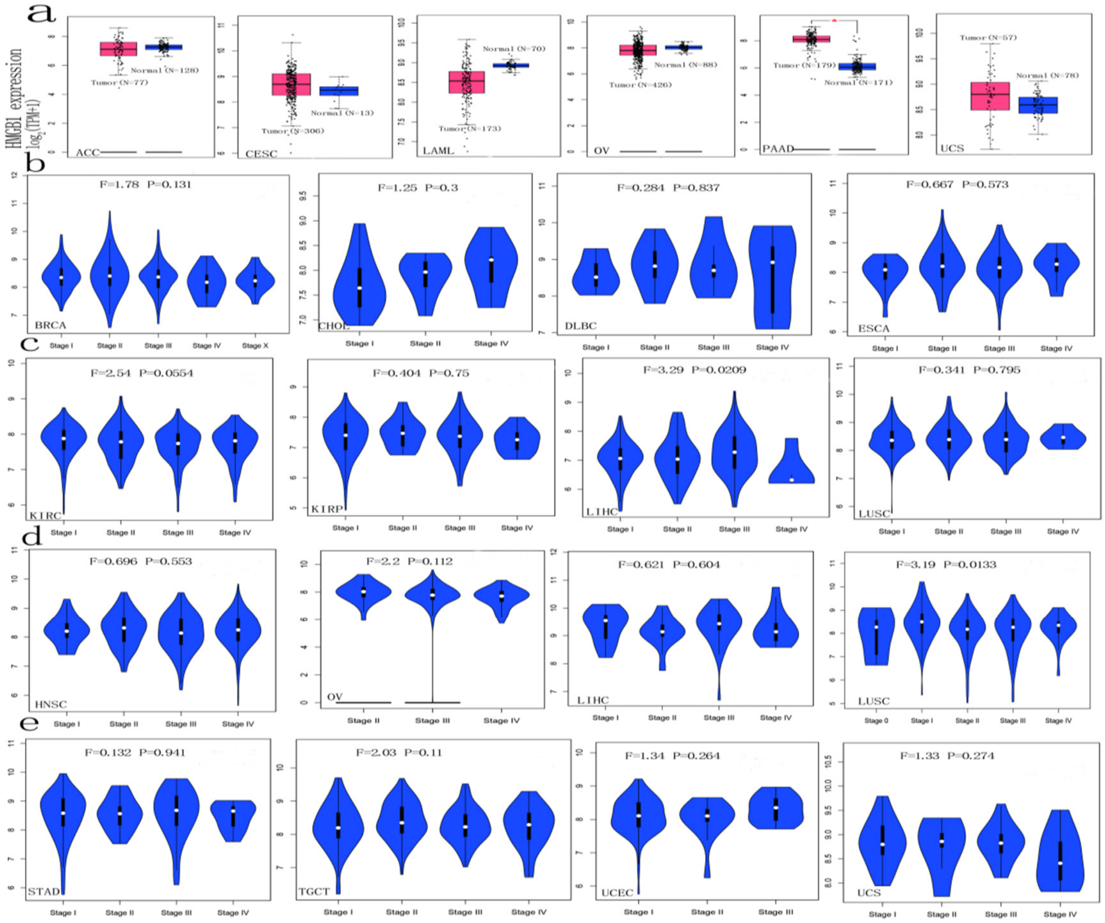

Figure S4. HMGB1 expression in certain cancers and pathological stages. (**a**) The box plots of HMGB1-related expression statuses in ACC, CESC, LAML, OV, PAAD, and UCS in TCGA database in normal tissues of the GTEx databases. (**b**) The violin plots of HMGB1 expression in different pathological stages of BRCA, CHOL, DLBC, ESCA (**c**) KIRC, KIRP, LIHC, LUSC (**d**) HNSC, OV, READ, SKCM (**e**) STAD, TGCT, UCEC, UCS.

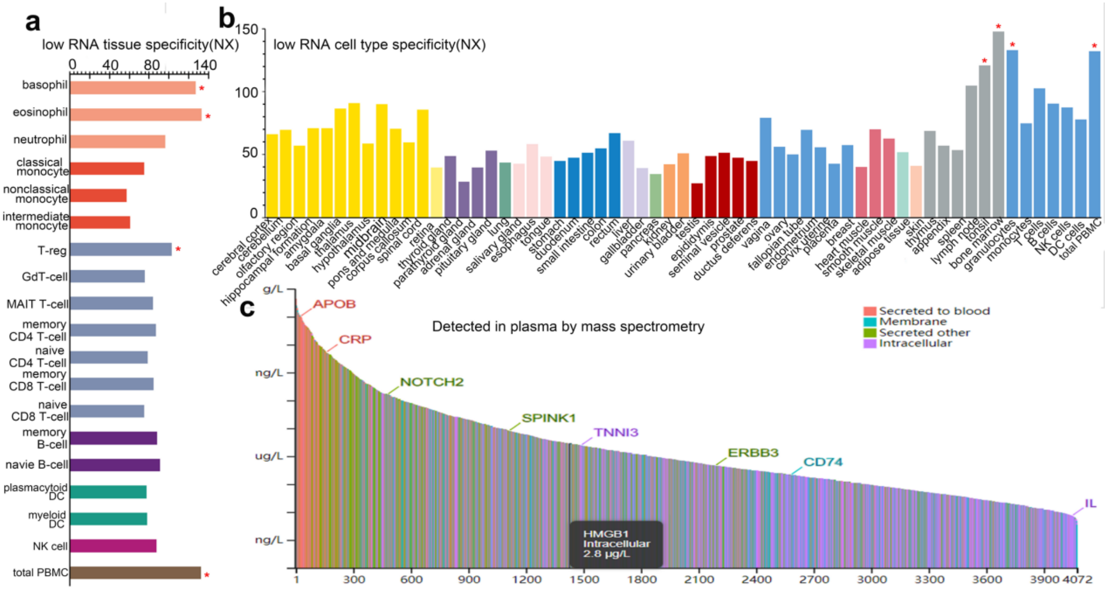

Figure S5. HMGB1 expression in different cell types, tissues and plasma in the normal physiological state. (**a**) The diagram of HMGB1expression in different tissues via the consensus datasets of HPA, GTEx and FANTOM5 (**b**) The diagram of HMGB1 expression in different tissues or blood

cells via the consensus dataset of HPA, Monaco and Schmiedel. (**c**) The diagram of HMGB1 gene in different tissues via the data of mass spectrometry.

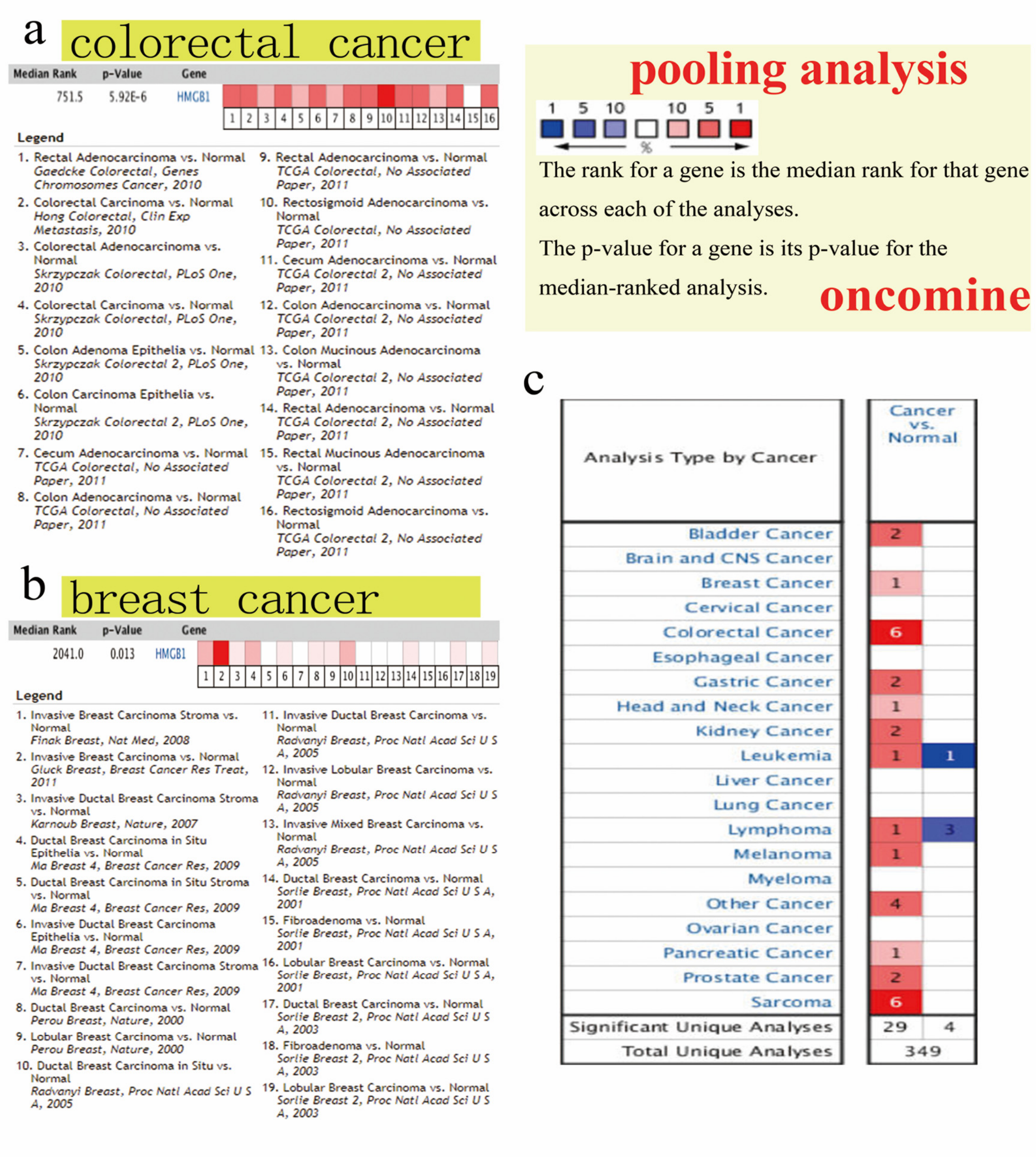

Figure S6. HMGB1 expression difference between normal and tumor tissues to pool the analysis via the Oncomine database. (**a**) Colorectal cancer (**b**) breast cancer (**c**) the analysis of HMGB1 expression with tumorous types.

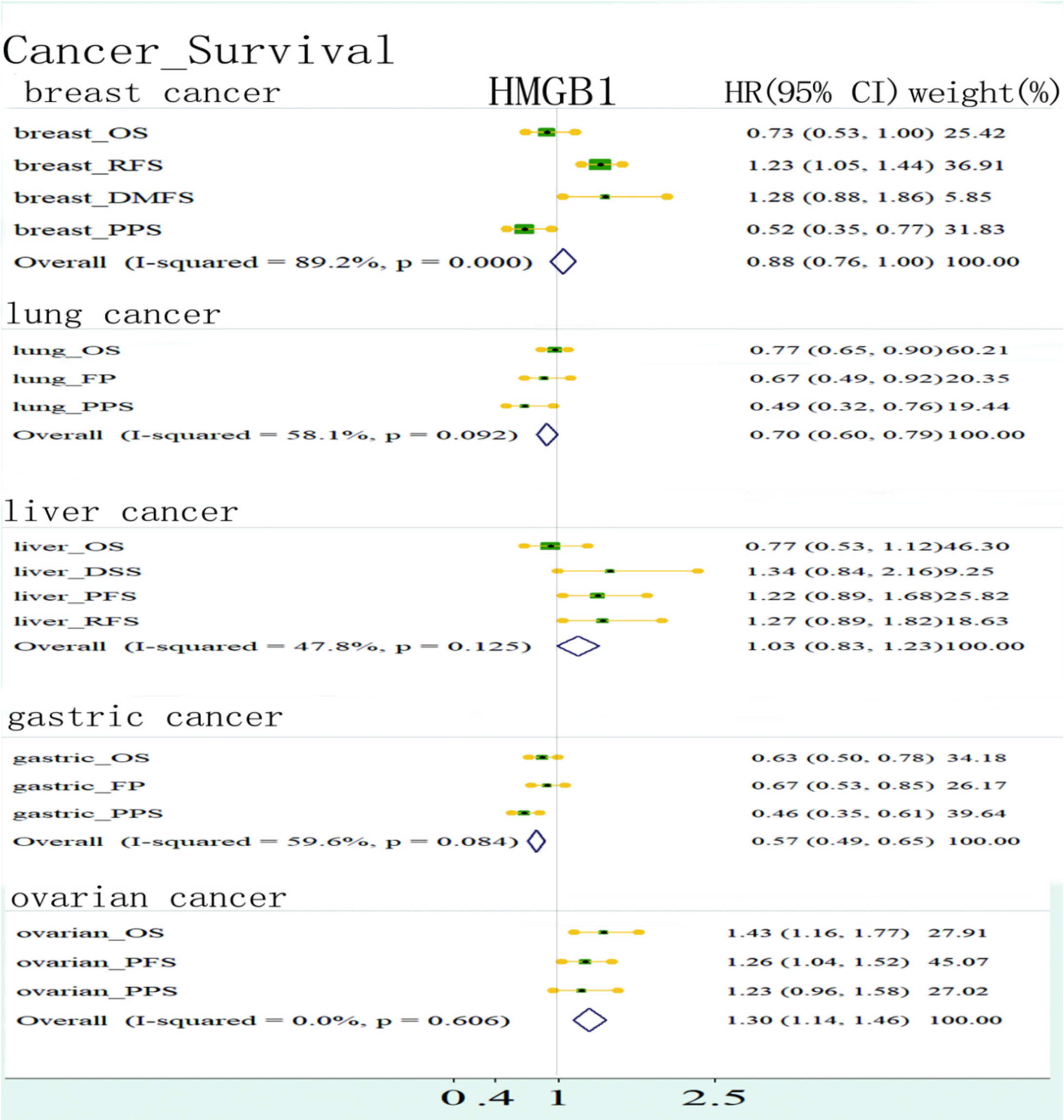

Figure S7. HMGB1 expression and prognosis of cancers via the Kaplan-Meier plotter. The HMGB1-related survival analyses of OS, DMFS, RFS, PFS, PPS, FP, and DSS in breast cancer, ovarian cancer, liver cancer, gastric cancer and lung cancer.

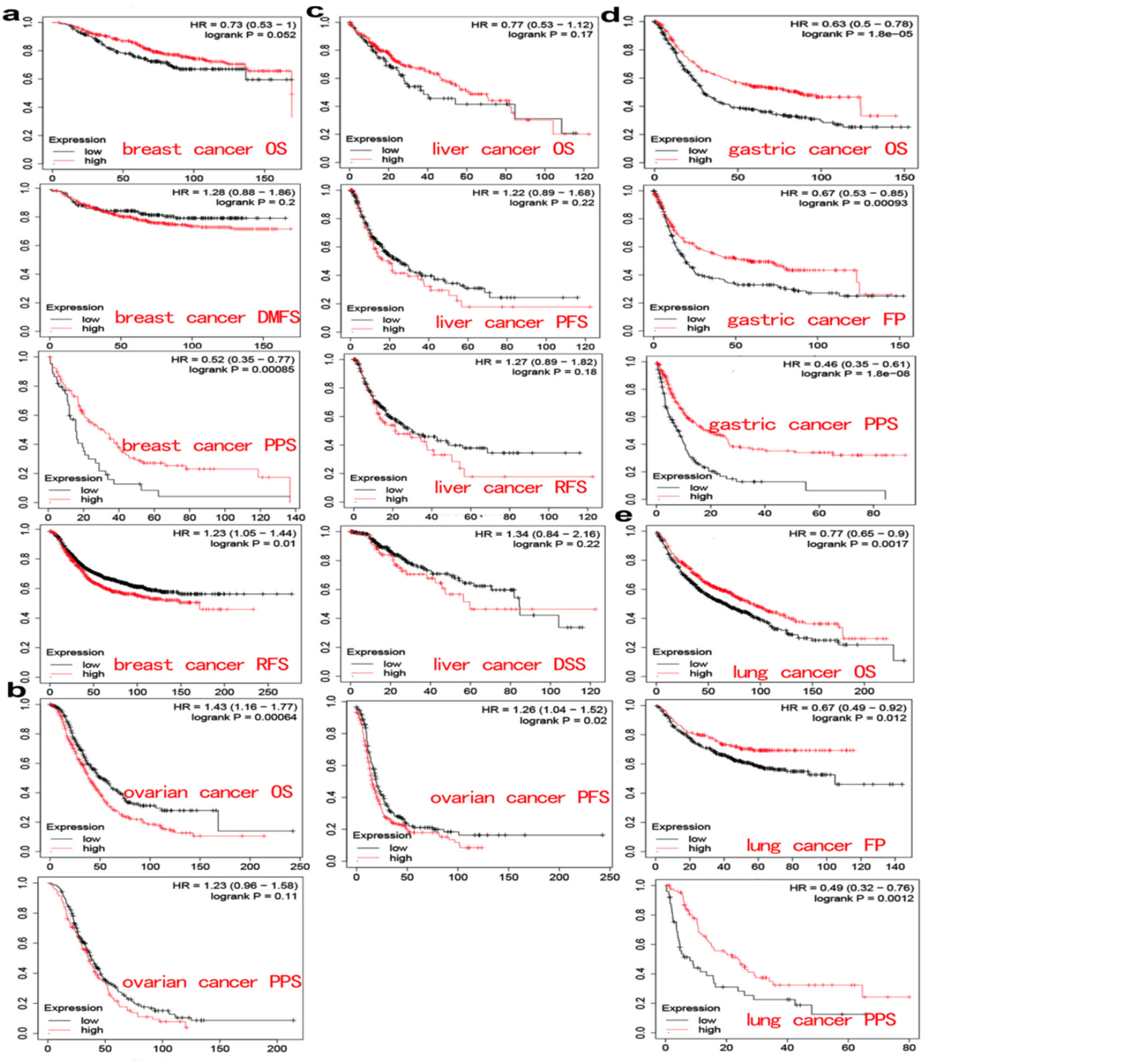

Figure S8. Meta-analysis on the correlation between HMGB1 expression and cancer prognosis. (**a**) breast cancer, (**b**) ovarian cancer, (**c**) liver cancer, (**d**) gastric cancer, and (**e**) lung cancer cases.

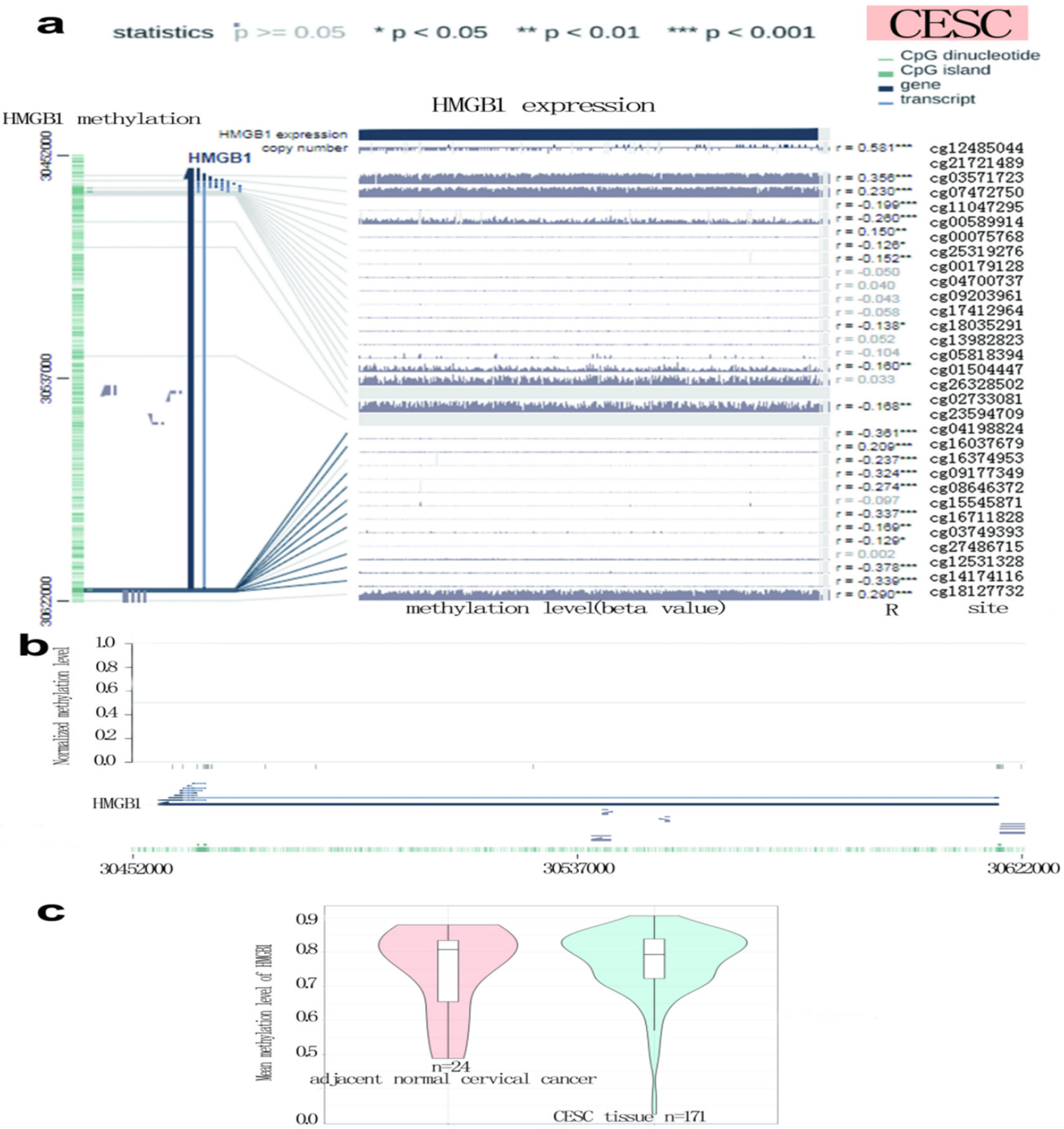

Figure S9. HMGB1-related DNA methylation and gene expression for the CESC cases of TCGA. (**a**) The diagram of HMGB1 relative multiple probes DNA methylation level via the MEXPRESS. The promoter region probes are highlighted. The beta value of methylation, the Benjamini-Hochberg-adjusted *p*-value and the Pearson correlation coefficients (R) are displayed. (**b**) Normalization of chip data (**c**) The violin plot of main methylation level of HMGB1 in the CESC tissues (n = 171) and adjacent normal tissues (n = 24).

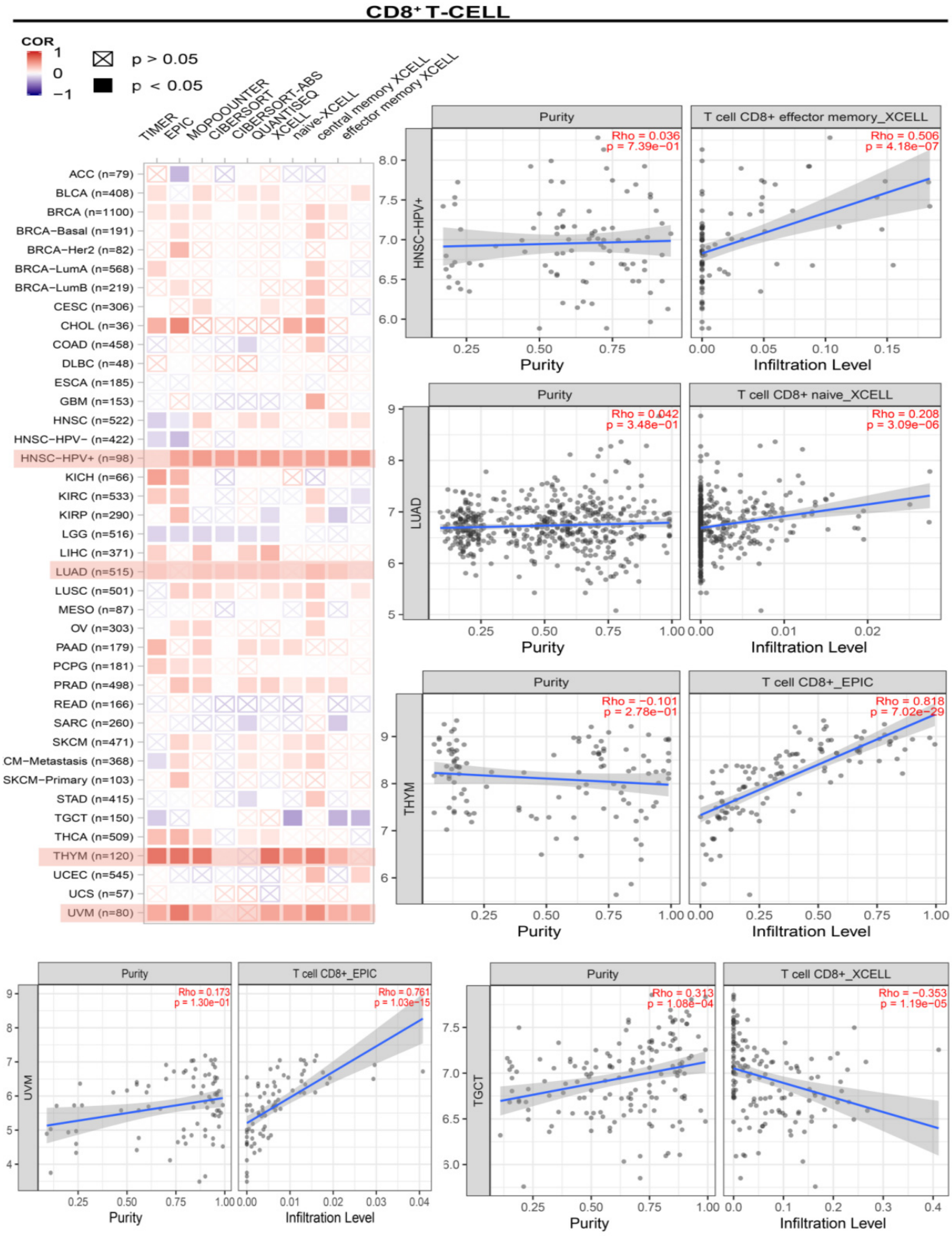

Figure S10. Correlation analysis between HMGB1 expression and immune infiltration of CD8⁺ T-cells in TCGA via different algorithms.

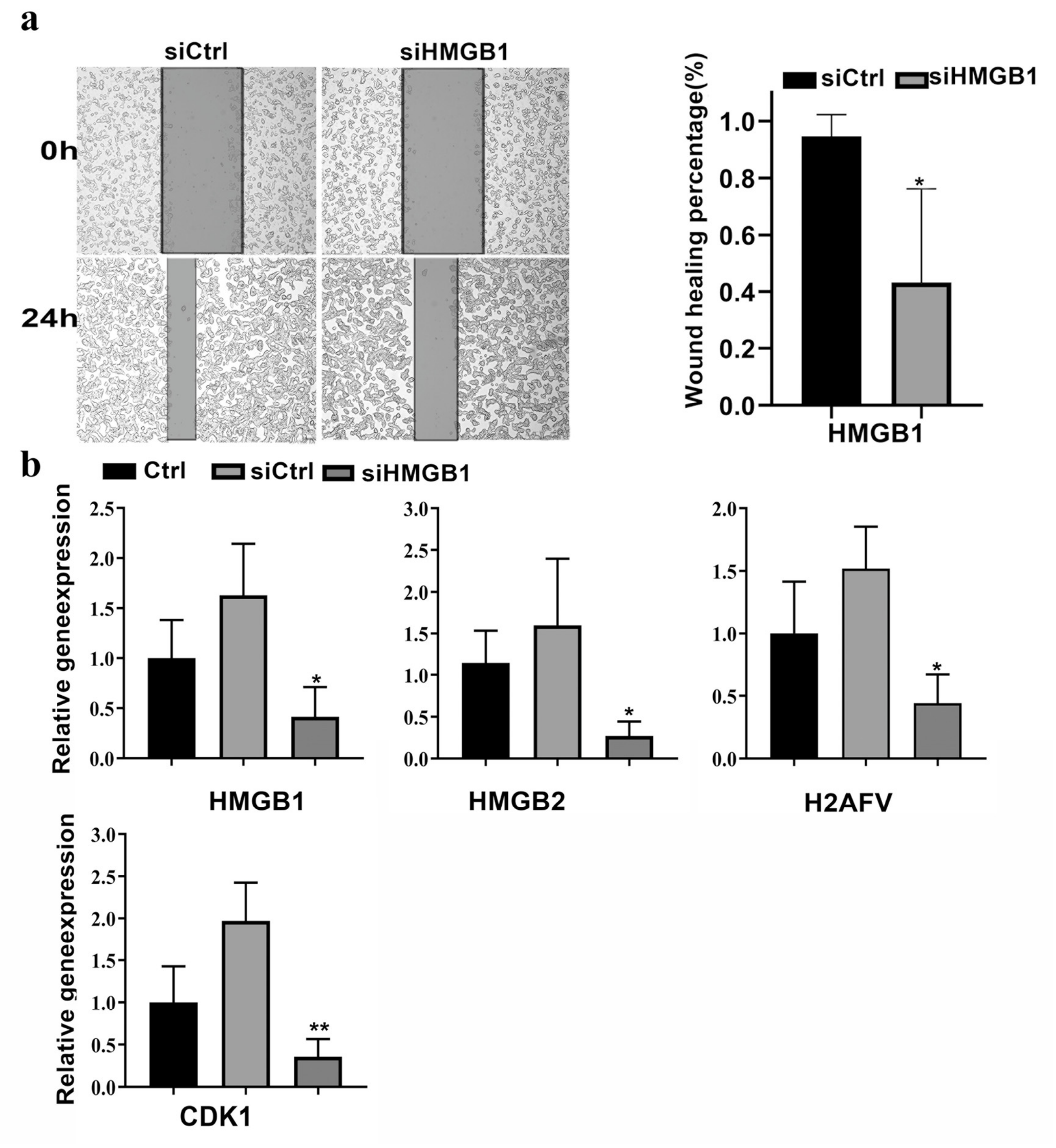

Figure S11. Functional validation of HMGB1 and expression of related in SW480 cell line. (**a**) Migration of SW480 cells assessed by wound healing assays. (**b**) HMGB1 knockdown reduces expression of its related gene including HMGB2, H2AFV, and CDK1 in SW480 cell line at gene expression level based on real time quantitative reverse transcriptase PCR (RT-qPCR).

Table S1. Correlative subgroup analysis of HMGB1 expression and prognosis of breast cancer cases.

Factor	subgroup	Sample size	OS		RFS		DMFS	
			HR	*p*	HR	*p*	HR	*p*
ER status	ER positive	2565	0.29	**0.014**	1.5	**0.0092**	0.47	0.1
	ER negative	1214	O.5	0.057	0.76	0.12	0.2	**0.015**
TP53 status	Mutated	232	0.61	0.48	1.73	0.075	2.71	0.055
	Wild type	363	NA	NA	0.32	**0.0082**	NA	NA
PR status	PR positive	954	NA	NA	0.71	0.09	0.4	0.11
	PR negative	1028	NA	NA	0.68	**0.039**	0.48	0.054
HER2 status	HER2 positive	416	5.36	**0.0077**	0.56	**0.034**	0.31	**0.031**
	HER2 negative	1456	0.34	0.087	1.63	**0.0024**	0.26	**0.023**
Grade	Grade1	378	0	**0.031**	3.15	0.11	NA	NA
	Grade2	1077	1.81	0.33	1.39	0.22	2.05	0.16
	Grade3	1090	0.64	0.09	1.46	**0.019**	1.69	0.087
Intrinsic subtype	Basal	879	1.33	0.39	1.42	**0.045**	2.1	**0.035**
	LuminalA	2504	0.54	**0.021**	1.21	0.14	1.46	0.25
	LuminalB	1425	0.51	**0.047**	1.56	**0.0048**	1.65	0.14
	$HER2^+$	335	0.68	0.4	0.7	0.21	0.58	0.16
Lymph node status	Lymph node positive	1459	0.56	0.084	0.74	**0.024**	0.59	0.093
	Lymph node negative	2259	2.11	0.13	1.71	**0.0064**	2.39	**0.044**
Pietenpol subtype	Basal-like1	239	NA	0.14	1.87	**0.035**	6.14	**0.014**
	Basal-like2	97	NA	**0.029**	3.81	**0.021**	4.62	0.11
	immunomodulatory	290	2.75	0.14	0.61	0.23	0.47	0.29
	Mesenchymal	229	2.67	0.18	2.25	**0.018**	2.37	0.15
	Luminal androgen receptor	276	0.36	**0.04**	0.68	0.24	0.35	**0.048**

HR, hazard ratio; OS, overall survival; RFS, relapse free survival; DMFS, distant metastasis free survival; ER, Estrogen receptor; PR, Progesterone receptor; HER2, human epidermal growth factor receptor-2; TP53, Tumor Protein P53; NA, not available data value less than 0.05 is shown in bold.

Table S2. Correlative subgroup analysis of HMGB1 expression and prognosis of lung cancer cases.

FACTOR	subgroup	Sample size	OS		FP		PPS	
			HR	*p*	HR	*p*	HR	*p*
Histology	adenocarcinoma	865	0.54	**7.1e−07**	0.53	**8.4e−05**	0.48	**0.0031**
	Squamous cell carcinoma	675	1.34	0.065	2.69	**0.00026**	2.06	0.26
Stage	Stage I	652	0.49	3.5e−05	0.5	**0.0054**	0.32	**7e−04**
	Stage II	320	0.65	0.13	1.97	**0.042**	0.47	**0.03**
	Stage III	70	1.68	0.14	NA	NA	NA	NA
gender	female	818	0.58	**0.013**	0.61	**0.028**	0.5	0.07
	male	1387	0.8	**0.034**	0.75	0.14	0.43	**0.0023**
Smoking history	exclude those never smoked	970	0.45	**0.00021**	1.3	0.27	0.51	**0.0074**
	Only those never smoked	247	0.33	**0.0042**	0.38	**0.0014**	0.51	0.16
AJCC stage t	Yes	178	0.35	0.11	0.51	0.21	0.21	**0.045**
	T1	475	1.38	0.23	0.47	0.28	NA	0.11
	T2	686	1.72	**0.005**	0.72	0.38	0.59	0.19
AJCC stage n	N0	863	0.74	0.09	0.41	**0.04**	1.56	0.29
	N1	296	1.79	0.02	2.32	0.06	0.61	0.46
AJCC stage m	M0	818	1.31	0.06	0.57	0.12	1.34	0.4
surgery	Only surgical margins negative	730	0.26	**0.00025**	0.39	**0.00061**	0.41	**0.017**

HR, hazard ratio; AJCC, American Joint Committee on Cancer; OS, overall survival; FP, first progression; PPS, post Progression survival; NA, not available data value less than 0.05 is shown in bold.

Table S3. Correlative subgroup analysis of HMGB1 expression and prognosis of ovarian cancer cases.

**FACTOR**	**subgroup**	**Sample size**	**OS**		**PFS**		**PPS**	
			**HR**	***p***	**HR**	***p***	**HR**	***p***
Histology	Endometrioid	62	0.56	0.61	1.97	0.24	NA	NA
	serous	1232	1.3	**0.024**	1.34	**0.011**	1.25	0.088
Stage	Stage I	107	0	0.071	0.29	0.097	NA	NA
	Stage II	72	0.22	**0.049**	1.59	0.31	0.23	0.16
	Stage III	1079	1.38	**0.012**	1.31	**0.031**	1.23	0.13
	Stage IV	189	0.77	0.37	1.33	0.28	1.72	0.13
TP53 mutation	Mutated	516	1.27	0.26	1.89	**0.00098**	0.81	0.27
	Wild type	102	0.63	0.4	2.8	**0.069**	0.72	0.54
Debulk	optimal	802	1.35	0.17	1.3	0.13	0.75	0.24
	suboptimal	536	1.39	**0.034**	1.55	**0.0018**	1.3	**0.094**
Grade	Grade 1	56	3.63	**0.023**	NA	**0.038**	NA	NA
	Grade 2	325	1.69	**0.025**	1.45	0.062	0.63	0.077
	Grade 3	1024	1.36	**0.019**	1.32	**0.028**	1.3	0.085
	Grade 4	21	0.57	0.26	NA	NA	NA	NA
Chemotherapy	Contains platin	1438	1.47	**0.0021**	1.48	**9.4e−05**	1.25	0.067
	Contains Taxol	821	1.46	**0.012**	1.51	**0.0013**	1.33	0.061
	ContainsTaxol + platin	804	1.46	**0.011**	1.51	**0.0014**	1.34	0.057

HR, hazard ratio; OS, overall survival; PFS, progress free survival; PPS, post progression survival; TP53, Tumor Protein P53; NA, not available data; *p*-value less than 0.05 is shown in bold.

Table S4. Correlative subgroup analysis of HMGB1 expression and prognosis of gastric cancer cases.

FACTOR	subgroup	Sample size	OS		FP		PPS	
			HR	*p*	HR	*p*	HR	*p*
Stage	Stage I	69	0.21	**0.0054**	0.48	0.25	0.18	**0.025**
	Stage III	319	0.49	**0.00019**	0.52	**0.00086**	0.33	**2.3e−07**
Gender	female	244	0.53	**0.0045**	0.51	**0.0018**	0.46	**0.009**
	male	567	0.61	**0.001**	0.71	**0.019**	0.57	**0.00082**
HER2	negative	641	0.67	**0.0044**	0.8	0.12	0.59	**0.0043**
	positive	425	0.65	0.054	0.6	**0.029**	0.5	**0.0065**
Differentiation	Poorly	166	0.57	**0.045**	0.5	0.0076	0.77	0.44
	Moderately	67	0.45	**0.033**	0.53	0.076	0.41	0.067
stage t	T2	253	0.69	**0.079**	0.67	0.068	0.67	0.077
	T3	208	0.58	**0.0031**	0.66	**0.022**	0.47	**0.00015**
stage n	N0	76	0.42	**0.045**	0.46	0.076	0.33	**0.046**
	N1	232	0.54	**0.0042**	0.62	**0.02**	0.48	**0.0016**
	N2	129	0.69	0.099	0.71	0.12	0.49	**0.0059**
stage m	M0	459	0.65	**0.0023**	0.69	**0.007**	0.61	**0.0012**
	M1	58	1.38	0.32	1.64	0.12	0.48	0.051
Lauren classification	Instestinal	336	0.52	**0.00088**	0.59	**0.0061**	0.45	**0.00021**
	Diffuse	248	0.69	**0.035**	0.73	0.075	0.7	0.067
Treatment	Surgery alone	393	0.65	**0.0037**	0.7	**0.017**	0.54	**0.00018**
	5-Fu based adjuvant	158	0.5	0.13	0.59	0.21	0.36	0.055
Perforation	No	169	0.51	**0.0037**	0.48	**0.00062**	0.72	0.24

HR, hazard ratio; OS, overall survival; FP, first progression; PPS, post progression survival; HER2, human epidermal growth factor receptor-2; NA, not available data; *p*-value less than 0.05 is shown in bold.

Table S5. Correlative subgroup analysis of HMGB1 expression and prognosis of liver cancer cases.

FACTOR	subgroup	Sample size	OS		PFS		RFS		DSS	
			HR	*p*	HR	*p*	HR	*p*	HR	*p*
Race	White	184	0.66	0.078	0.66	**0.038**	0.71	0.15	0.61	0.086
	Asian	158	0.7	0.27	1.47	0.16	1.37	0.23	1.99	0.085
Stage	Stage I	171	0.62	0.16	1.39	0.2	1.63	0.075	1.67	0.26
	Stage II	86	0.72	0.42	1.41	0.29	1.39	0.33	2.06	0.19
	Stage III	85	0.74	0.34	0.64	0.12	0.61	0.16	0.76	0.51
gender	female	121	1.18	0.56	0.75	0.3	0.7	0.26	0.79	0.53
	male	250	0.68	0.11	1.41	0.077	1.51	0.051	1.63	0.097
Vascular invasion	None	205	0.66	0.13	0.8	0.34	1.36	0.23	1.91	0.075
	micro	93	0.39	0.073	1.35	0.32	1.77	0.17	0.19	0.078
Grade	Grade 1	55	1.89	0.18	1.97	0.095	2.08	0.14	2.7	0.091
	Grade 2	177	0.5	**0.012**	0.67	0.086	0.67	0.12	0.39	**0.0052**
	Grade 3	122	0.83	0.54	1.28	0.33	1.31	0.33	1.66	0.19
Sorafenib treatment	treated	30	0.11	**1e−04**	0.33	**0.0071**	0.35	**0.024**	0.11	**1e−04**
AJCC _T	T1	181	0.58	0.085	1.36	0.22	1.59	0.085	0.63	0.26
	T2	94	0.64	0.23	1.51	0.2	1.5	0.28	0.44	0.088
	T3	80	1.38	0.32	0.57	0.057	0.59	0.11	0.81	0.59
Alcohol consumption	Yes	117	0.61	0.14	0.41	**0.0014**	0.41	**0.0051**	0.51	0.075
	none	205	1.57	0.07	1.4	0.098	1.4	0.14	2.09	**0.02**
Hepatitis virus	yes	153	0.68	0.28	1.17	0.5	1.27	0.35	0.78	0.56
	none	169	1.6	0.054	1.32	0.23	1.47	0.14	1.94	**0.027**

HR, hazard ratio; AJCC, American Joint Committee on Cancer; OS, overall survival; FP, first progression; PPS, post progression survival; HER2, human epidermal growth factor receptor-2; NA, not available data; *p*-value less than 0.05 is shown in bold.

Table S6. Prediction of CPTAC-identified phosphorylation sites of HMGB1 based on the PhosphoNET.

site	sequence	Experimentally confirmed#	Hydrophobicity	p-site similarity score	Maximum kinase specificity	Sum kinase specificity score	Conservati-on score
**T22**	SYAFFVQTCREEHKK	NA	−1.033	−63.8	127	3,943	36.4
**S35**	KPRGKMSSYAFFVQT	NA	−0.633	−61.3	493	17,057	33.6
**S39**	PDASVNFSEFSKKCS	NA	−0.813	−59.6	356	15,114	20.6
**S42**	SVNFSEFSKKCSERW	NA	−1.187	−63.0	299	12,824	43.0
**T78**	YEREMKTYIPPKGET	NA	−1.600	−57.8	609	26,836	33.9
**S100**	NAPKRPPSAFFLFCS	NA	−0.107	−60.6	449	20,166	0.0
**Y109**	FFLFCSEYRPKIKGE	20068231	−0.333	−60.1	349	16,073	18.8
**S121**	KGEHPGLSIGDVAKK	20068231	−0.800	−57.5	320	13,019	37.0
**Y162**	YEKDIAAYRAKGKPD	20068230	−1.427	−56.9	373	16,381	38.8

#The PMID (PubMed Unique Identifier) information of the publication was provided; NA, not available.

Table S7. The following primers were presented.

Name	Primer	Sequence (5'–3')
HMGB1	Forward Primer	TCTCAGGGCCAAACCGATAG
	Reverse Primer	TCGTGCACCGAAAGTTTCAA
HMGB2	Forward Primer	CCAGGCAAGAATACCCTCCA
	Reverse Primer	CCGCGAAATTGACGGAAGAG
H2AFV	Forward Primer	GCCGCATCCACAGACACTT
	Reverse Primer	TACCTGCCAGCTCCAGCA
CDK1	Forward primer	CGTAGCTGGGCTCTGATTGG
	Reverse primer	TGGTAGATCCGCGCTAAAGG
ACTB	Forward primer	CACCATGGATGATGATATCGC
	Reverse primer	CATAGGAATCCTTCTGACCCA
